# Public health approaches to children affected by parental incarceration: the central role of school nursing in supporting a vulnerable population

**DOI:** 10.1177/17449871261419695

**Published:** 2026-02-26

**Authors:** Sarah Bekaert

**Affiliations:** Associate Professor, Oxford School of Nursing and Midwifery, Oxford Brookes University

School nurses are uniquely positioned to support children affected by parental incarceration, yet these children remain a substantial but largely hidden group within health, education, and social care. Despite growing political attention to this concern in the United Kingdom, many such children continue to go unidentified and without appropriate support. This paper outlines the national scale and urgency of the issue, examines the emotional, developmental and social impacts on children who experience parental incarceration, and considers how schools can act as protective environments. Drawing on key theoretical frameworks, it highlights the cumulative effects of the associated adversity, and the gaps in current identification and support processes. The central argument advanced is that school nurses, working within the Healthy Child Programme, can provide early intervention, sustained therapeutic oversight, and coordinated multiagency support. The paper concludes by calling for systematic national recognition of these children as a vulnerable group and for a continued systematic, well-resourced model of care, including specialist school nursing roles.

## Scale and urgency of the issue

Up to 310,000 children in England and Wales are affected by parental incarceration each year (Kincaid et al., 2019), yet they remain largely invisible within health and education systems. Although data on prisoners who are parents is not routinely collected, with prison numbers rising – 85,851 in June 2023 and projected to exceed 94,000 by 2025 – the number of affected children is expected to grow accordingly (Sturge and Tunnicliffe, 2023). Whilst third-sector organisations provide valuable therapeutic support, equitable provision requires a national, systematic public health pathway for children affected by parental incarceration. Formal recognition of children who experience parental incarcerations as a vulnerable group, and automatic allocation to a health professional with developmental, safeguarding and therapeutic expertise, is essential. School nursing services, positioned at the interface of health and education, are ideally placed to deliver this support (Bekaert, 2025).

## The impact of parental incarceration on children

Children with a parent in prison face a complex mix of emotional, developmental, and practical challenges. These may include disrupted attachment, grief, anxiety, guilt and confusion, alongside increased responsibilities at home (Bates and Bayles, 2015; Boswell and Wedge, 2002). Older children, particularly adolescents, may be more vulnerable to substance use and risk-taking behaviours as coping mechanisms (Shlafer and Poehlmann, 2010). School attendance and behaviour can deteriorate, increasing the risk of exclusion, academic underachievement, and poorer long-term outcomes (Leeson and Morgan, 2022).

Parental incarceration constitutes an ‘ambiguous loss’– a form of grief characterised by the ongoing presence of a parent who is physically absent (Boss, 2010 in Levkovich and Ne’emani, 2022). This leaves the situation unresolved and confusing as finality and integration of the loss into the child’s life is denied. Further emotions such as guilt, loneliness, anxiety, shame, concern for the parents, withdrawal, and fearfulness have been noted (Levkovich and Ne’emani, 2022; Wilbur et al., 2007). Such childhood emotional and cognitive challenges can lead to neurodevelopmental challenge and health and behavioural consequences (Arditti and Savla, 2015). Trauma and chronic stress can cause physical health problems such as migraines and asthma (Nosek et al., 2019), as well as disrupted sleep and eating behaviours (Jackson and Vaughn, 2017). There is also increased likelihood of depression (Swisher and Roettger, 2012). Although on occasion decreased depression may occur – likely linked to the relief and reprieve from ongoing stressors caused by the incarcerated parent (Swisher and Roettger 2012; Wilbur et al., 2007). Attention-deficit hyperactivity disorder (ADHD; Nosek et al., 2019), aggression (Wilbur et al., 2007) and ‘anti-social’ behaviour (Swisher and Roettger 2012) are greater for children where a parent is in prison. There is also an increased possibility of youth offending, and being imprisoned themselves in later life (Wilbur et al., 2007).

Furthermore, the dynamics of intrafamilial relationships are impacted through parental incarceration which, in turn, may have a negative impact on the child. For example, a reduction in parental oversight, due to the absence of a parent and/or the remaining parent under increased pressure and less able to monitor the child or young person’s activity (Swisher and Roettger, 2012). There may be practical changes incurred by a sudden shift in material circumstances such as needing to relocate to a new house, live with another family member, or enter foster care (Correa et al., 2021; Song et al., 2018).

The loss of parental presence, extra pressure on the remaining parent, disruption to home life, and stigma of having a parent in prison can negatively affect a child’s social inclusion. Such stigma can play its part in the child or young person’s disengagement with school and other community groups. Young people who have experienced parental incarceration report exclusionary treatment by their peer group, sometimes to the point of physical and emotional violence (Arditti and Savla, 2015). Unsurprisingly this can result in silencing, and the decreased likelihood of children and young people seeking or accepting support (Nosek et al., 2019). Mistrust of authoritarian organisations can also be a factor leading to withdrawal from potentially supportive organisations and accepting support from professionals. Disconnection and disengagement with school specifically leads to children and young people missing out on the positive social and academic benefits of the school community (Nichols et al., 2016).

The incarceration of a parent is also unlikely to be an isolated event or the beginning of a story. The child or young person may have experienced challenging situations and events over time, and possibly exposed to criminal or negative behaviours such as family violence or drug use. Such families are vulnerable before the parent’s incarceration, and these vulnerabilities change and/or increase after imprisonment (Correa et al., 2021). Parental incarceration can often be chronic, causing re-traumatisation and cumulative considerations (Wilbur et al., 2007).

## Theoretical perspectives

Understanding the impact of parental incarceration through established theoretical frameworks underpins the need for a coordinated response led by skilled health professionals. Attachment theory highlights how sudden separation from a parent, regardless of the quality of the relationship, can undermine secure attachments (Bowlby, 1969; Ainsworth et al., 1978). Children and young people with insecure attachment may be more likely to develop behavioural challenges (Theule et al., 2016) and can have difficulty forming healthy relationships in adolescence and adulthood (Miga et al., 2010). In addition, developmental theory, such as cognitive development (Piaget, 1952) and psychosocial development (Erikson, 1968), emphasise that children’s needs and responses vary by age. For example, adolescents may be particularly vulnerable due to their developmental stage and greater exposure to risk, and the cumulative impact of parental incarceration may only become apparent later in life.

Parental incarceration generates economic, relational, and emotional pressures for the family. Stress plays out across the family network through interdependence among family members (Song et al., 2018). This is articulated through stress or strain theory (Agnew, 1992, 2020) and the family stress model (Hill, 1958). Parental incarceration is recognised as an adverse childhood experience (ACE), associated with disrupted emotional development, family instability and increased risk of negative health and social outcomes (Gjelsvik et al., 2014; Turney, 2018). Resilience theory highlights the protective role of positive childhood experiences (PCEs), which include stable relationships, supportive schools, positive role models, and access to community resources (Bethell et al., 2019; Han et al., 2023). Where parental incarceration can weaken these supports through loss of social capital, increased isolation and disruption to daily routines – resilience refers to achieving positive developmental outcomes despite significant adversity (Riley and Masten, 2005). Feeling connected across multiple contexts, particularly home and school, is essential to positive development (Nichols et al., 2016). When family social capital is compromised, schools can play a crucial mediating and protective role for affected children.

## The protective role of schools

In the United Kingdom, the Farmer Review (Ministry of Justice, 2017) highlights the protective role of schools as safe, supportive environments for children, particularly those affected by parental incarceration. School nurses, as specialist public health practitioners, are well-placed to identify and support these children. They occupy an embedded yet professionally independent position within schools: commissioned by Local Authorities as part of 0–19 public health services rather than employed by schools. This organisational separation allows them to maintain professional autonomy, provide confidential, child-centred support, and address public health priorities across both school and community settings (Local Government Association, 2022a, 2022b).

The Healthy Child Programme (Office for Health Improvement and Disparities [OHID], 2023) is a public health framework that provides universal and targeted health promotion, screening and early intervention services to support the health, development, and well-being of children from pregnancy through adolescence. School nurses work within the Healthy Child Programme and contribute to safeguarding, early intervention, health promotion, assessment, and multiagency coordination. Trained in child development, family-centred care, trauma-informed practice, and therapeutic communication, school nurses provide emotional support, assess physical and mental health, and identify emerging risks. Acting as a bridge between school, family and community services, they ensure children have access to mental health support, early help, youth services and specialist voluntary organisations, promoting resilience and well-being. Their expertise ideally positions them as the named professional supporting children affected by parental incarceration.

## The gap: lack of identification and systematic support

There is currently no formal national mechanism for identifying children affected by parental incarceration, and prisons do not routinely notify local services when a parent is incarcerated. As a result, many children remain unidentified until problems such as absence, behavioural issues or mental health difficulties emerge. Working with such vulnerable groups of young people reflects the premise of the aims of the ‘No child left behind’ guidance from Public Health England (2020) to support vulnerable children and young people and mobilise protective factors which enable children to prosper despite experiencing vulnerability or adversity.

To address this gap, Thames Valley Police, in partnership with Children Heard & Seen – a third-sector organisation supporting children with incarcerated parents – launched Operation Paramount in 2021. This first-of-its-kind scheme automatically identifies children whose parent has been sent to prison using prison data, rather than relying on self-referral. Identified families are offered tailored support, including mentoring, peer support, legal and financial advice and social activities, to mitigate the harm of parental incarceration. Evaluation indicates that these children experience higher levels of disadvantage, lower school attendance, and increased support needs (Olphin et al., 2025). The programme has demonstrated effectiveness in early identification and intervention, helping to reduce the negative impacts of parental incarceration (Bekaert and Raju, 2025; Bekaert et al., 2025). A toolkit has been developed to guide other areas considering similar initiatives (Bekaert and Massie, 2025); however, there is still a need for a national directive to formalise recognition and support for children affected by parental incarceration.

While third-sector specialist support is essential, there is a need to embed consideration of parental incarceration within statutory provision, creating a systematic and formally accountable process. Incorporating this into the Healthy Child Programme (OHID, 2023) would formally recognise school nurses as the practitioners best placed to oversee support for affected children. Once identified, school nurses can undertake a holistic assessment of the child’s emotional well-being, physical health, developmental needs, caregiving responsibilities, financial pressures, school engagement, and wider family context. This enables the coordination of targeted, multiagency support tailored to the child’s needs, ensuring consistent, accountable intervention across education, and health and social care systems.

Currently, there is very little guidance or policy structure to assist school nurses in supporting children with a parent in prison, compared with systems available for other vulnerable groups. Leeson and Morgan (2022) suggested extending the eligibility criteria for young carers to include children with an incarcerated parent. The Farmer Review (Ministry of Justice, 2017) identifies these children as young carers and argues that their needs should be recognised and addressed in a non-judgemental, non-stigmatised manner. School nurses already play an established role in supporting young carers (Local Government Association, 2022b; Lovell and Cleaver, 2015). A range of existing frameworks, including the Children’s Society guides (2017, 2018) for identifying, supporting and signposting young carers, could therefore be adapted to provide structured guidance and processes for children affected by parental incarceration.

## The need for additional resources and specialist roles

For school nurses to deliver this enhanced role effectively, investment is essential. Workforce shortages and large caseloads currently limit capacity for sustained therapeutic work (Bekaert and Sutton, 2024). Recognising parental incarceration within national policy as a key area of vulnerability, with school nurses identified as the most appropriate specialist practitioners to support these children, must be accompanied by increased funding for school nursing teams, dedicated training in trauma and the criminal justice system, and manageable caseloads.

A creative solution could be the creation of School Nurses with a Special Interest (SNSI) in children who experience parental incarceration. Similar to specialist roles in other branches of nursing, these practitioners would hold advanced expertise, oversee complex cases, lead local pathways, liaise with prisons and visitor centres, provide supervision to colleagues and contribute to regional and national policy development. Such roles would also offer a clear career pathway within school nursing, enhancing retention and professional development (Bekaert et al., 2025).

## Towards a national, systematic and preventative approach

Embedding a support offer for children and families affected by parental incarceration within national health policy would transform current fragmented support. Systematic recognition and allocation to school nurses would ensure no child remains hidden, enabling early, coordinated intervention and sustained specialist oversight. With dedicated resources, manageable caseloads, and potential SNSI, this approach would reduce health inequalities, mitigate long-term, harm and promote resilience, giving children affected by parental incarceration the best chance to thrive despite adversity.

**Key points for policy, practice and/or research**

- Children with a parent in prison face emotional, social, and developmental challenges, including anxiety, behavioural difficulties, and disrupted family life.
- Schools provide a protective environment, and school nurses can offer holistic, child-centred support, safeguarding, and multiagency coordination.
- Many children remain unidentified due to lack of formal mechanisms, though initiatives like Operation Paramount show the value of early recognition.
- Young carers’ frameworks could be adapted, and specialist school nursing roles could provide expertise, oversee complex cases, and lead support pathways.
- A national, systematic approach with dedicated resources and formal recognition would ensure no child is overlooked, enabling early, coordinated support and promote resilience.
